# The prognostic significance of apoptosis-associated proteins BCL-2, BAX and BCL-X in clinical nephroblastoma

**DOI:** 10.1054/bjoc.2001.2146

**Published:** 2001-11

**Authors:** M A Ghanem, T H Van der Kwast, J C Den Hollander, M K Sudaryo, M M Van den Heuvel, M A Noordzij, R J M Nijman, E H Soliman, G J van Steenbrugge

**Affiliations:** Dept. of Paediatric Urology and Pathology, Josephine Nefkens Institute; The Netherlands Institute for Health Sciences, Erasmus University; Dept. Paediatric Oncology, University Hospital, Rotterdam, The Netherlands; Urology Dept., Menoufiya University, Egypt

**Keywords:** Wilms’, tumour, Bcl-2, Bax, Bcl-X, prognosis

## Abstract

Apoptotic cell death represents an important mechanism for the precise regulation of cell numbers in normal tissues. Various apoptosis-associated regulatory proteins, such as Bcl-2, Bax and Bcl-X, may contribute to the rate of apoptosis in neoplasia. The present study was performed to evaluate the prognostic value of these molecules in a group of 61 Wilms' tumours of chemotherapeutically pre-treated patients using an immunohistochemical approach. Generally, Bcl-2, Bax and for Bcl-X _S/L_ were expressed in the blastemal and epithelial components of Wilms' tumour. Immunoreactive blastema cells were found in 53%, 41% and 38% of tumours for Bcl-2, Bax and for Bcl-X _S/L_, respectively. An increased expression of Bcl-2 was observed in the blastemal component of increasing pathological stages. In contrast, a gradual decline of Bax expression was observed in the blastemal component of tumours with increasing pathological stages. Also blastemal Bcl-X _S/L_ expression decreased with stage. Univariate analysis showed that blastemal Bcl-2 expression and the Bcl-2/Bax ratio were indicative for clinical progression, whereas epithelial staining was of no prognostic value. Multivariate analysis showed that blastemal Bcl-2 expression is an independent prognostic marker for clinical progression besides stage. These findings demonstrate that alterations of the Bcl-2/Bax balance may influence the clinical outcome of Wilms' tumour patients by deregulation of programmed cell death.   http://www.bjcancer.com © 2001 Cancer Research Campaign


*British Journal of Cancer* (2001) **85**(10), 1557–1563
© 2001 Cancer Research Campaign
doi: 10.1054/ bjoc.2001.2146, available online at http://www.idealibrary.com on IDEAL

http://www.bjcancer.com

### The prognostic significance of apoptosis-associated proteins BCL-2, BAX and BCL-X in clinical nephroblastoma

**MA Ghanem[1], TH Van der Kwast[2], JC Den Hollander[2], MK Sudaryo[3], MM Van den Heuvel[4], MA Noordzij[1], RJM Nijman[1], EH Soliman[5] and GJ van Steenbrugge[1]**

[1]Dept. of Paediatric Urology and [2]Pathology, Josephine Nefkens Institute; [3]The Netherlands Institute for Health Sciences, Erasmus University; [4]Dept. Paediatric Oncology, University Hospital, Rotterdam, The Netherlands; [5]Urology Dept., Menoufiya University, Egypt

**Summary** Apoptotic cell death represents an important mechanism for the precise regulation of cell numbers in normal tissues. Various apoptosis-associated regulatory proteins, such as Bcl-2, Bax and Bcl-X, may contribute to the rate of apoptosis in neoplasia. The present study was performed to evaluate the prognostic value of these molecules in a group of 61 Wilms' tumours of chemotherapeutically pre-treated patients using an immunohistochemical approach. Generally, Bcl-2, Bax and for Bcl-$X_{S/L}$ were expressed in the blastemal and epithelial components of Wilms' tumour. Immunoreactive blastema cells were found in 53%, 41% and 38% of tumours for Bcl-2, Bax and for Bcl-$X_{S/L}$, respectively. An increased expression of Bcl-2 was observed in the blastemal component of increasing pathological stages. In contrast, a gradual decline of Bax expression was observed in the blastemal component of tumours with increasing pathological stages. Also blastemal Bcl-$X_{S/L}$ expression decreased with stage. Univariate analysis showed that blastemal Bcl-2 expression and the Bcl-2/Bax ratio were indicative for clinical progression, whereas epithelial staining was of no prognostic value. Multivariate analysis showed that blastemal Bcl-2 expression is an independent prognostic marker for clinical progression besides stage. These findings demonstrate that alterations of the Bcl-2/Bax balance may influence the clinical outcome of Wilms' tumour patients by deregulation of programmed cell death. © 2001 Cancer Research Campaign http://www.bjcancer.com

**Keywords**: Wilms' tumour; Bcl-2; Bax; Bcl-X; prognosis

Apoptosis, a genetically programmed mechanism of eliminating cells in response to a variety of stimuli, is a widespread phenomenon occurring at different stages of morphogenesis, growth and development as well as in normal turnover of adult tissues (Reed, 1994). Loss of apoptotic response in tumour cells may be one of the mechanisms involved in malignant progression and resistance to chemotherapy (Arends and Wyllie, 1991). Studies have shown that several proteins are involved in the regulation of apoptosis. Abnormalities in the expression of these proteins may contribute to tumorigenesis by altering the balance between cell growth and cell death (Vaux, 1993). A number of cellular and viral homologues of Bcl-2 have been identified. Beyond Bcl-2 this family of proteins includes Bcl-X, and Mcl-1 which act as blockers of apoptosis, whereas others such as Bax and Bak appear to promote apoptosis (Oltvai et al, 1993).

Structurally, Bcl-2 is an integral membrane protein localised to membranes of the nucleus and endoplasmic reticulum. The protein product of the Bax gene, which has 21% homology to the Bcl-2 protein, can form heterodimers with Bcl-2 leading to abrogation of its ability to suppress apoptosis (Oltvai et al, 1993). In its long splice variant (Bcl-$X_L$), Bcl-X is another potent antagonist of apoptosis, whereas the short splice variant (Bcl-XS) promotes cell death. Recent studies demonstrated that Bcl-$X_L$ is mainly involved in the mechanism of resistance to chemotherapeutic agents and radiation (Datta et al, 1995). An abnormal pattern of Bcl-2, Bax and Bcl-X expression has been demonstrated in several human malignancies (McDonnel et al, 1992; Gazzaniga et al, 1996; Krajewska et al, 1996a,b; Xie et al, 1998).

Wilms' tumour, or nephroblastoma, is a paediatric malignancy of the kidney and one of the most common solid tumours in children (Beckwith, 1994). Preoperative chemotherapy as treatment for Wilms' tumour has increasingly been more used in Europe by International Society of Paediatric Oncology (SIOP) which is different from the protocol used in the USA by the National Wilms' Tumour Study (NWTS) (D'Angio, 1983). Despite the remarkable response to chemotherapy, 5% to 10% of tumours are fatal due to metastases and/or the occurrence of drug resistance (Green et al, 1994). Since it appears that programmed cell death is a crucial event in normal kidney development one may expect oncogenic events to result from defects in the pathways of renal cell death (Coles et al, 1993). Therefore, the prognostic value of Bcl-2, Bax and for Bcl-$X_{S/L}$ were investigated in specimens of nephroblastoma patients which were chemotherapeutically treated prior to surgery. Having access to this type of material derived from a substantial number of patients with a good stage distribution, the aim of our study was to find factors which could predict the clinical outcome of these patients after being treated by chemotherapy and surgery.

*Received 11 September 2000*
*Revised 31 August 2001*
*Accepted 31 August 2001*

*Correspondence to:* GJ van Steenbrugge

### MATERIALS AND METHODS

### Patients

During the period 1987–1999, 61 patients with nephroblastoma were treated by neo-adjuvant chemotherapy and subsequent tumour nephrectomy. Following treatment patients were followed regularly and all data concerning diagnosis, treatment and follow-up were stored in a database. Clinical progression was defined as histologically or cytologically proven local recurrence or the appearance of distant metastases.

### Sample selection

All nephrectomy specimens were fixed in 10% buffered formalin and embedded in paraffin. The haematoxylin and eosin-stained slides were reviewed by an experienced paediatric pathologist (JCDH). The tumour stage (pT stage) was assigned according to adaptation of the SIOP trial protocol established in the SIOP meeting in Stockholm in 1994 (Boccon-Gibod, 1998). Among the paraffin blocks available of individual patients those containing tissue with the 3 different cell types of Wilms' tumour (blastemal, epithelial and stromal) were selected. In addition, adjacent normal kidney tissue was taken from each patient.

### Antibodies

The following primary antibodies were used: mouse monoclonal antibody against Bcl-2 (clone 124) (DAKO, A/S, Glostrup, Denmark) and rabbit anti-human Bax (P-19) and Bcl-$X_{S/L}$ (S-18 against both the Bcl-$X_L$ and Bcl-$X_S$) polyclonal antibodies from Santa Cruz, California, USA. The specificity and characteristics of these antibodies have been published elsewhere (Oltvai et al, 1993; Datta et al, 1995).

### Immunohistochemistry

The PAP (peroxidase-anti-peroxidase) technique was used. Serial sections (5 µm) from all samples were mounted on 3-aminopropyl-trietoxysilane (Sigma Co, St Louis, MO, USA) coated glass slides, which were incubated overnight in a 60˚C incubator. After dewaxing in fresh xylene for 10 min and passage in 100% methanol for 10 min, the sections were rinsed in methanol containing 3% hydrogen peroxide for 20 min to block endogenous peroxidase activity. The slides were rinsed with distilled water. In order to enhance antigen exposure, the slides were microwaved at 700 W in 0.1 M citrate buffer at pH 6.0 for 15 min. After cooling and rinsing with PBS, the slides were placed in a Sequenza immunostaining system (Shandon, Uncorn, UK). Sections were incubated with 10% normal rabbit serum (prior to monoclonal antibody) or 10% normal goat serum (prior to polyclonal antibody) (DAKO). Incubation was done in PBS/5% bovine serum albumin (BSA) for 15 min and subsequently overnight with the primary antibody at 4˚C. The antibodies were diluted in PBS/5% BSA at 1:50 for Bcl-2, 1: 150 for Bax and 1:75 for Bcl-$X_{S/L}$. Subsequently, the slides were rinsed with PBS/Tween (0.1%) and incubated for 30 min with rabbit-anti-mouse antibody for monoclonal antibody or goat-anti-rabbit for polyclonal antibody (DAKO), followed by rinsing with PBS/Tween (0.1%). The PAP complex (DAKO) was diluted in PBS/5% BSA at 1:300 and incubated for 30 min followed by rinsing with PBS. The antigen-antibody binding was visualised with diaminobenzidine tetrahydrochloride dihydrate (Fluka, Neu-Ulm, Germany) as chromogen. The sections were lightly counterstained with haematoxylin. Negative controls were included by replacing the primary antibody by PBS/5% BSA. Normal kidney tissue adjacent to the tumour served as positive control.

### Immunostaining analysis

The slides were examined at 25 × magnification without knowledge of the clinical outcome of the patients. The amount of positive staining was assessed as the estimated percentage positively staining cells: < 10% was considered negative, > 10% was considered positive.

### Statistical analysis

Statistical analysis was performed using the SPSS 9 software package. The association between Bcl-2, Bax, Bcl-$X_{S/L}$ expression and clinico-pathological features was analysed using $\chi^2$ test. For analysis of survival data Kaplan–Meier curves were constructed and the log-rank test was performed. Multivariate analysis was performed using Cox's proportional hazards model with $P < 0.05$ considered statistically significant.

### Protein extraction and Western blot analysis

To further confirm the Bcl-$X_{S/L}$ immunohistochemical data, Western analysis was performed with tissues from Wilms' tumour xenografts. 6 different xenograft tissues were analysed in total. Tissues 1–3 (cf. Figure 4) originate from transplants of three individual patients, resulting in xenografts WT-7, WT-9 and WT-11, respectively, whereas tissues 4–6 (WT-15, WT-15LN, WT-16) were from one individual patient, being a specimen of primary tumour in right kidney (WT-15), lymph node metastasis (WT-15LN), and primary tumour in left kidney (WT-16), respectively. Morphologically, all 6 tissues contained the blastemal and stromal component, whereas in tissue 1 and 3 epithelial cells were present as well. The frozen tissues were crushed in a liquid nitrogen chilled metal cylinder. The tissue homogenates were transferred to a lysis buffer consisting of 10 mM TRIS (pH 7.4), 150 mM NaCl (Sigma), 1% triton × 100 (Merck, Darmstadt, Germany), 1% deoxycholate (Sigma), 0.1% sodium dodecyl sulfate (SDS, Gibco BRL), 5 mM EDTA (Merck) and protease inhibitors (1 mM phenylmethyl-sulfonyl fluoride, 1 mM aprotinin, 50 mg $l^{-1}$ leupeptin, 1 mM benzamidine and 1 mg $l^{-1}$ pepstatin, all from Sigma). The samples were spun at 35 000 ***g*** at 4˚C for 10 minutes. The protein content of the supernatant was measured photometrically using the bio-rad protein assay (Bio-rad, München, Germany). The proteins were transferred to a SDS-polyacrylamide gel and electrophoresis was performed in 10 times diluted tray buffer for 2 hours. The gel was blotted to a 0.45 µm cellulose nitrate membrane (Scheicher & Schuell, Dassel, Germany). Pre-stained markers were used as size standards (Novex, San Diego, CA). The immunoblot was blocked for 1 hour with 5% dry milk (Sigma) in 0.1% Tween-20 (Sigma). The antibodies were diluted 1:1000 in 5% dry milk and applied overnight at 4˚C. After rinsing with PBS/0.1% Tween, the blot was incubated with horseradish peroxidase-labelled goat-anti-rabbit (1:2000, DAKO) for 1 hour. Subsequently, a one minute incubation with a 1:1 mixture of luminol and oxidizing reagent (DuPont NEN, Chemiluminescence kit, Boston, MA) was performed. Excess reagent was removed by placing the blot on a piece of Whatmann paper. Finally, the antibodies were visualised by exposure of the blot to an X-ray film for 30 seconds.

 © 2001 Cancer Research Campaign

### RESULTS

### Clinico-pathological findings

The tumour-stage distribution was $T_1$ in 21, $T_2$ in 20 and $T_3$ in 20 patients. There were 29 (48%) females and 32 (53%) males. The mean age at surgery was 4.2 years, and the mean overall follow-up period was 5.7 years. Clinical progression occurred in 14 patients (23%), and 8 patients (13%) died from their tumour. At the end of the follow-up period 53 patients were alive.

### BCL-2, BAX, BCL-$X_{S/L}$ expression in normal renal tissues

Immunoreactivity for Bcl-2 was found in the Henle's loop, collecting ducts and parietal layer of Bowman's capsule of the normal kidney (Figure 1A). Immunoreactivity for Bax was found in the proximal and distal convoluted tubules and parietal layer of Bowman's capsule, but was not expressed in the collecting ducts (Figure 1B). Expression of Bcl-$X_{S/L}$ was evident mainly in the collecting duct and to lesser extent in the proximal and distal convoluted tubules, whereas expression in the glomeruli was not found (Figure 1C).

### BCL-2 expression in Wilms' tumour tissues

Bcl-2 positive (i.e. > 10% stained) blastemal and epithelial cells were found in 32 (53%) and 33 (54%) of the Wilms' tumours studied, respectively (Table 1) Bcl-2 staining was mainly perinuclear, but some cytoplasmic staining was also found (Figure 1D). Some speci-mens showed expression of the majority of cancer cells, whereas in

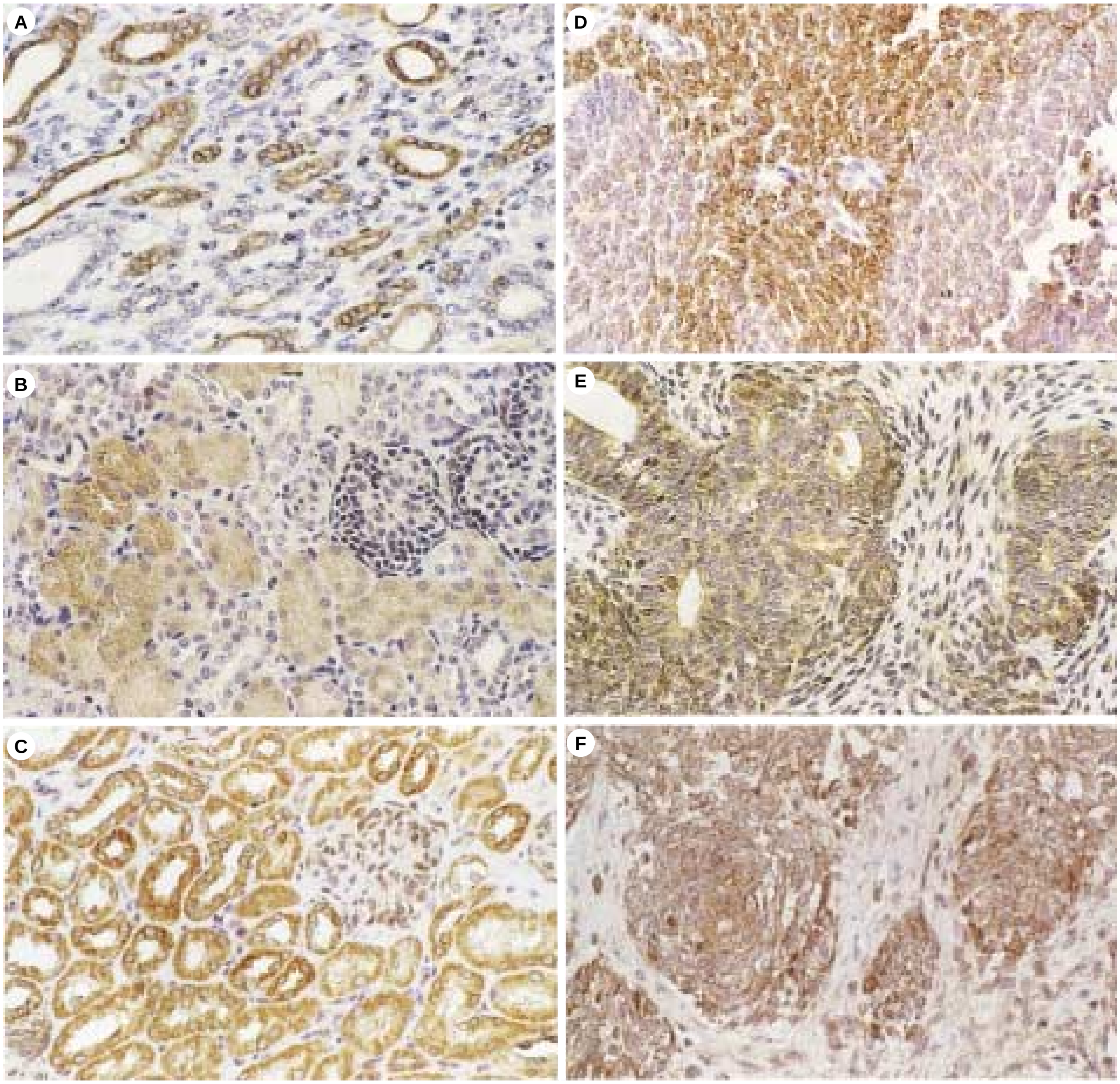

**Figure 1** Normal renal tissue sections and nephroblastoma tissues were immunostained for Bcl-2 (**A**, **D**); Bax (**B**, **E**); Bcl-$X_{S/L}$ (**C**, **F**), respectively. Slides were counterstained with haematoxylin (magnification 400 ×)

*© 2001 Cancer Research Campaign*

**Table 1** Relationship between pT stage and blastemal and epithelial expression

| Stage | Bcl-2 | | Bax | | Bcl-$X_{S/L}$ | |
|---|---|---|---|---|---|---|
| | Blastema | Epithelial | Blastema | Epithelial | Blastema | Epithelial |
| T1 | 9 (15) | 9 (15) | 10 (16) | 10 (16) | 9 (15) | 13 (21) |
| T2 | 11 (18) | 12 (20) | 9 (15) | 10 (16) | 8 (13) | 13 (21) |
| T3 | 12 (20) | 12 (20) | 6 (10) | 7 (11) | 6 (10) | 9 (15) |
| Total | 32 (53) | 33 (54) | 25 (41) | 27 (44) | 23 (38) | 35 (57) |
| *P* value | > 0.05 | | > 0.05 | | > 0.05 | |

Data are presented as numbers, percentages between brackets.

**Table 2** Univariate analysis of prognostic markers

| Variable | Blastemal | | | | Epithelial | | | |
|---|---|---|---|---|---|---|---|---|
| | Clinical progression | | Tumour-specific survival | | Clinical progression | | Tumour-specific survival | |
| | $\chi^2$ | *P** | $\chi^2$ | *P** | $\chi^2$ | *P** | $\chi^2$ | *P** |
| Bcl-2 | 7.31 | **0.006** | 3.32 | 0.07 | 0.01 | 0.94 | 1.09 | 0.29 |
| Bax | 1.11 | 0.29 | 1.02 | 0.31 | 1.79 | 0.19 | 0.26 | 0.61 |
| Bcl-$X_{S/L}$ | 0.52 | 0.47 | 1.22 | 0.27 | 1.44 | 0.23 | 0.63 | 0.43 |
| Bcl-2/Bax | 10.31 | **0.02** | 4.56 | 0.21 | 1.92 | 0.59 | 1.33 | 0.72 |

*Log-rank test.

others only small areas of the tumour were found positive for the Bcl-2 protein. The majority of the infiltrating lymphocytes in the tumour stroma were strongly Bcl-2 positive, serving as internal control for the staining procedure. Bcl-2 expression gradually increased from $T_1$, $T_2$ to $T_3$ for both the blastemal and epithelial component. No statistically significant relationship between stage and Bcl-2 expression was found. The nephroblastoma sections showed an intense expression of the epithelium with little expression in the stromal component.

### BAX expression in Wilms' tumour tissues

Blastemal and epithelial expression of Bax was found in a similar percentage of Wilms' tumours (41 and 43% of cells, respectively) (Table 1). Bax staining was mainly cytoplasmic (Figure 1E) and the staining pattern of most of the specimens was weak to moderate. Bax expression in both blastema and epithelium gradually decreased from $T_1$ to $T_3$, but the trend was not statistically significant. The epithelial component of the tumour demonstrated diffuse expression of Bax.

### BCL-$X_{S/L}$ expression in Wilms' tumour tissues

Blastemal and epithelial expression of Bcl-$X_{S/L}$ was found in 23 (38%) and 35 (57%) of the Wilms' tumour studied, respectively (Table 1). The percentage of tumours with Bcl-$X_{S/L}$-positive blastema was generally lower than those staining positive for Bcl-2. The majority of Bcl-$X_{S/L}$-positive tumours had very intense cytoplasmic staining pattern, showing a more widely distribution (Figure 1F). There was no statistically significant relationship between stage and Bcl-$X_{S/L}$ expression.

Immunoblot analysis of tissue lysates of a panel of human Wilms' tumour xenografts identified the specificity of the Bcl- $X_{S/L}$ antibody used for immunohistochemistry (Figure 4). Morphologically, all 6 tissues contained blastemal and stromal component, whereas in tissue 1 and 3 epithelial cells were present as well. The Bcl-$X_L$ protein was found to have migrated as a doublet of proteins with an apparent molecular mass of 28–32 kDa. The Bcl-$X_L$ was clearly detectable in all 6 samples analysed, particularly the 32 kDa message. A clear 22 kDa band compatible with Bcl-$X_S$ was detected in 3 samples positive for Bcl-$X_L$, whereas faint bands were found in the remaining 2 samples, of which one was devoid of Bcl-$X_S$ (Figure 4).

### Prognostic value of BCL-2, BAX and BCL-$X_{S/L}$

Univariate analysis using the logrank test showed a prognostic value of blastemal Bcl-2 expression for clinical progression (Table 2, Figure 2). The blastemal expression for both Bax and Bcl-$X_{S/L}$ and epithelial expression of the 3 proteins did not show any prognostic value (Table 2). To test whether Bcl-2, Bax and Bcl-$X_{S/L}$ expression had any prognostic impact a multivariate Cox' regression analysis was done including the parameters pT stage, Bcl-2,

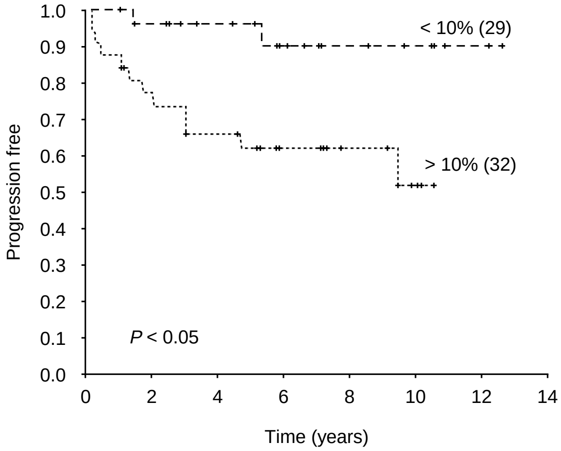

**Figure 2** Kaplan–Meier curves showing the relationship between blastemal Bcl-2 expression and clinical progression. Censored patients are indicated by tic marks along their line. Number of patients per group is shown between brackets

 *© 2001 Cancer Research Campaign*

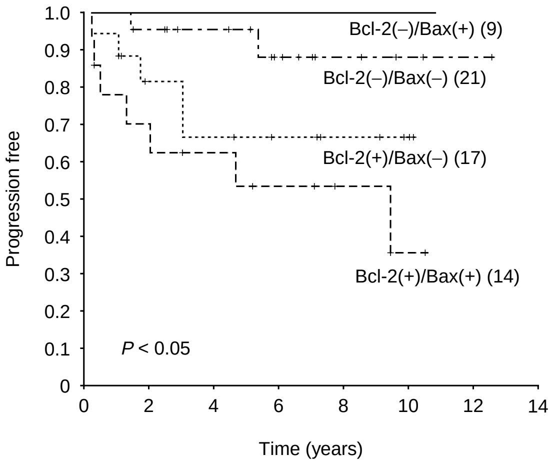

**Figure 3** Kaplan–Meier curves showing the relationship between clinical progression and blastemal ratio of Bcl-2/Bax. Censored patients are indicated by tic marks along their line. Number of patients per group is shown between brackets

Bax and Bcl-$X_{S/L}$ expression. PT stage was divided as pT1-2 vs. pT3. In that analysis, only the Bcl-2 blastemal expression could be identified as independent prognostic variable besides stage (data not shown).

### Prognostic value of the Bcl-2/Bax ratio

By examining the Bcl-2 to Bax ratio versus outcome, 4 patients groups were identified. The Kaplan–Meier curves of Figure 3 show the prognostic influence of Bcl-2/Bax expression on the time to progression. Clearly, patients with high (>10%) expression of both Bcl-2 and Bax had a statistically significant poor prognosis compared to the 2 groups of patients with Bcl-2- negative expression (i.e., Bcl-2 and Bax negative or Bcl-2 negative/Bax positive) (Table 2, Figure 3). Generally, the data of Figure 3 is consistent with the notion that the patients with low Bcl-2/Bax ratio do better than those with high ratio. Bcl-2 and Bax were simultaneously expressed in 28% and 25% of blastemal and epithelial components of tumours, respectively.

### DISCUSSION

Over the past few years, Bcl-2 has moved from being a molecule exclusively implicated in lymphoid translocation (14; 18) to being an important gene involved in key mechanisms in the regulation of cell death, by inhibiting apoptosis. Consequently, Bcl-2 is implicated in the process of multidrug resistance of cancer. For this reason, studying the prognostic value of Bcl-2 gene family in malignant processes is warranted. In the present study we have examined the expression of Bcl-2 and some of its related proteins, Bax and Bcl-$X_{S/L}$, to study its prognostic value in Wilms' tumour. All patients studied received chemotherapy before operation. It is realised that in the majority of patients having a good response upon chemotherapy, treatment did affect the cellular compartments of the Wilms' tumour, the blastema in particular. Consequently, this may influence the (Bcl-2, Bax, and Bcl-$X_{S/L}$) immunophenotype of the remaining tumour component. The present study did not allow comparative studies of the effect of treatment upon the expression of the apoptotic markers studied. The expression level of the various markers, however, reflects the status of the tumour after chemotherapy, regardless of the occurrence of drug resistance. Therefore the final status of the tumour removed, may predict the clinical outcome of the patient, e.g. whether metastasis will occur.

Expression of Bcl-2, Bax and Bcl-$X_{S/L}$ was found in normal kidney tissues, which were used as internal positive controls for the immunohistochemical reaction (Figure 1A, B, C) as well as for the Western blot (Figure 4). In general, increased Bcl-2 expression was related to advanced disease. Reduction of Bax immunostaining in high-stage tumours inconsistent with the more aggressive character of these tumours. Most clinically oriented studies on Bcl-2 expression have found a positive correlation with adverse prognosis in a variety of solid tumours, including non-small-cell lung carcinoma (Pezzella et al, 1993), nasopharyngeal carcinoma (Lu et al, 1993), prostatic carcinoma (McDonnel et al, 1992), and neuroblastoma (Castle et al, 1993). In contrast, Bcl-2 expression was correlated with favourable prognosis in tumours arising from epithelial as in stage II colon carcinoma (Sinicrope et al, 1995) and node-positive treated breast cancer (Gasparini et al, 1995). However, in other studies of patients with renal cell tumours (Paraf et al, 1995), invasive transitional cell carcinoma of the bladder (Glick et al, 1996), and head and neck cancer (Drenning et al, 1998), such correlation was not found. A recent study failed to demonstrate prognostic Bcl-2 significance in Wilms' tumour because of a limited number of cases studied (Re et al, 1999). In another study, Tanaka et al (1999) showed that preoperative chemotherapy did not significantly influence either the occurrence of apoptosis or expression of Bcl-2 in a group of treated Wilms' tumour patients. In the present study, a prognostic value of blastemal Bcl-2 expression was found for clinical progression. The mechanism by which Bcl-2 contributes to the progression of nephroblastoma is unknown. Based on gene transfer experiments (Vaux et al, 1988) and studies of transgenic mouse models (Strasser et al, 1990), Bcl-2 functions by inhibiting cell death rather than affecting the rate of cell proliferation.

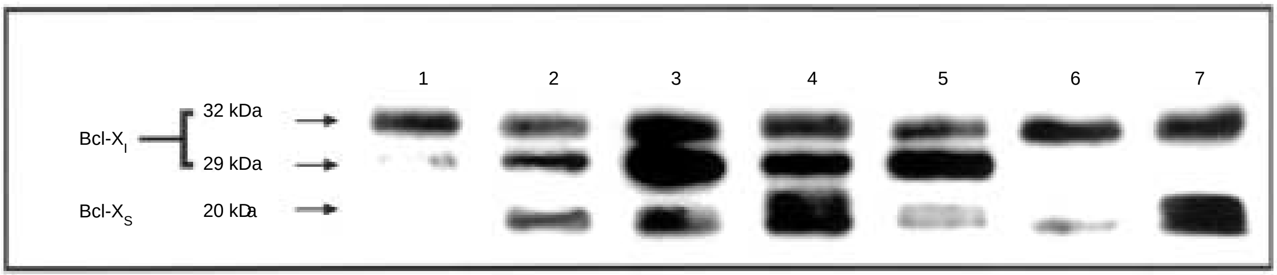

**Figure 4** Immunoblot analysis of Bcl-$X_{S/L}$ proteins in nephroblastoma tissues derived of 6 human xenografts models (lanes 1–6) and normal kidney (lane 7) with approximate indication of size marker

Information regarding the prognostic significance of Bax in human tumours is scarce. It has, however, been shown that reduced Bax expression correlates with poor prognosis in patients treated with chemotherapy for metastatic breast adenocarcinomas (Krajewski et al, 1995) and in radiotherapeutic glottic squamous cell carcinomas (Xie et al, 1998). High levels of Bax expression correlates to a better outcome for patients with low-grade tumour of urinary bladder (Gazzaniga et al, 1996). Bax expression alone had no influences upon survival of stage I patients with radically resected non-small-cell lung cancer (Apolinario et al, 1997), however, a study of the Bax mRNA expression levels in nephroblastoma, was not indicative that its expression could have a role in the prognosis (Re et al, 1999). This was confirmed by the outcome of the present study showing an absence of any prognostic value of Bax. The ratio between the Bcl-2 and the Bax proteins has been described as a cellular rheostat determining the cellular response to apoptotic stimuli (Oltvai et al, 1993). Given the potential role of these proteins in malignant tumours, the prognostic significance of combined Bcl-2 and Bax expression has been studied by many authors. Gazzaniga et al (1996) demonstrated that the Bcl-2/Bax expression ratio had prognostic impact for disease progression in low-grade bladder tumours. Interestingly, in nephroblastoma Bcl-2/Bax expression had prognostic significance for the prediction of clinical progression at univariate analysis.

The present data demonstrate that the level of Bcl-2 expression exceeds that of Bcl-$X_{S/L}$, although Bcl-$X_{S/L}$ staining is extensive and intensive compared to Bcl-2. These results are not similar to those reported for untreated prostatic and gastric cancer, in which Bcl-$X_{S/L}$ immunoreactivity was found in 100 and 85% of the tumours, respectively (Krajewska et al, 1996a,b). Bcl-$X_{S/L}$ was expressed in 100% of untreated gliomas, but not related to the response to radiotherapy or chemotherapy (Rieger et al, 1998). In the present study, Western blot analysis demonstrated that the anti-apoptotic Bcl-$X_L$ protein was the dominant isoform of the Bcl-$X_{S/L}$ protein in experimental human nephroblastoma. This was in agreement with the outcome of studies of Re et al suggesting the oncogenic potential of Bcl-$X_L$ overexpression in the development of anaplastic Wilms' tumour (Re et al, 1999). Accordingly, reduction of Bcl-$X_L$ expression may influence the outcome of patients treated with radiotherapy and/or chemotherapy (Datta et al, 1995; Krajewska et al, 1996b; Decaudin et al, 1997). On the basis of the present non-quantitative immunohistochemical data of various apoptosis-associated proteins no conclusion can be drawn with respect to the clinical behaviour of Wilms' tumours. Still, reduction of the expression of the anti-apoptotic Bcl-$X_L$ protein, may contribute to enhanced sensitivity to radiotherapy/chemotherapy. Indeed, cumulating in vitro data suggest that Bcl-$X_L$ may play an important role in the susceptibility of tumour cells to chemotherapeutic agents and radiation. For this experimental study it seems that the immunohistochemical expression of one single marker will provide sufficient information on the chemosensitivity or radiosensitivity.

In conclusion, Bcl-2, Bax and Bcl-$X_{S/L}$ were expressed in normal renal tissues and in nephroblastoma. Bcl-2 expression and the Bcl-2/Bax ratio showed prognostic value in the specimens of chemotherapeutically treated Wilms' tumour patients.

### ACKNOWLEDGEMENTS

We wish to thank Mr Rejiv B Mathoera for his valuable help and Mr Frank van der Panne for photographical assistance.

### REFERENCES

Apolinario RM, van der Valk P, de Jong JS, Deville W, van Ark-Otte J, Dingemans AM, van Mourik JC, Postmus PE, Pinedo HM and Giaccone G (1997) Prognostic value of the expression of p53, bcl-2, and bax oncoproteins, and neovascularization in patient with radically resected non-small-cell lung cancer. *J Clin Oncol* **15**: 2456–2466

Arends MJ and Wyllie AH (1991) Apoptosis: mechanisms and roles in pathology. *Int Rev Exp Pathol* **32**: 223–254

Beckwith JB (1994) Renal neoplasms of childhood. In: Sternberg SS (ed.) *Diagnostic Surgical Pathology*. pp 1741–1766. New York: Raven Press

Boccon-Gibod L (1998) Pathological evaluation of renal tumors in children: International society of pediatric oncology approach. *Ped Dev Pathol* **1**: 243–248

Castle VP, Heidelberger KP, Bromberg J, Ou X, Dole M and Nunez G (1993) Expression of the apoptosis-suppressing protein bcl-2, in neuroblastoma is associated with unfavoarable histology and N-myc amplification. *Am J Pathol* **143**: 1543–1550

Coles HS, Burne JF and Raff MC (1993) Large-scale normal cell death in the developing rat kidney and its induction by epidermal growth factor. *Development* **118**: 777–784

D'Angio GJ (1983) SIOP and the managment of Wilms' tumour. *J Clin Oncol* **1**: 595–596

Datta R, Manome Y, Taneja N, Boise LH, Weichselbaum R, Thompson CB, Slapak CA and Kufe D (1995) Overexpression of Bcl-XL by cytotoxic drug exposure confers resistance to ionizing radiation-induced internucleosomal DNA fragmentation. *Cell Growth Differ* **6**: 363–370

Decaudin D, Geley S, Hirsch T, Castedo M, Marchetti P, Macho A, Kofler R and Kroemer G (1997) Bcl-2 and Bcl-$X_L$ anatgonize the mitochondrial dysfunction preceding nuclear apoptosis induced by chemotherapeutic agents. *Cancer Res* **57**: 62–67

Drenning SD, Marcovitch AJ, Johnson DE, Melhem MF, Tweardy DJ and Grandis JR (1998) Bcl-2 but not Bax expression is associated with apoptosis in normal and transformed squamous epithelium. *Clin Cancer Res* **4**: 2913–2921

Gasparini G, Barabareschi M, Doglioni C, Palma PD, Mauri FA, Boracchi P, Bevilacqua P, Caffo O, Morelli L, Verderio P, Pezzella F and Harris AL (1995) Expression of bcl-2 protein predicts efficacy of adjuvant treatments in operable node-positive breast cancer. *Clin Cancer Res* **1**: 189–198

Gazzaniga P, Gradilone A, Vercillo R, Gandini O, Silvestri I, Napolitano M, Albonici L, Vincenzoni A, Gallucci M, Frati I and Agliano AM (1996) Bcl-2/Bax mRNA expression ratio as prognostic factor in low-grade urinary bladder cancer. *Int J Cancer* **69**: 100–104

Glick SH, Hawell LP and White RW (1996) Relationship of p53 and bcl-2 to prognosis in muscle invasive transitional cell carcinoma of bladder. *J Urol* **155**: 1754–1757

Green DM, Beckwith JB, Breslow NE, Faria P, Moksness J, Finklestein JZ, Grundy P, Thomas PR, Kim T and Schochat S (1994) Treatment of children with stages II to IV anaplastic Wilms' tumor: a report from the National Wilms' Tumor Study Group. *J Clin Oncol* **12**: 2126–2131

Krajewska M, Fenoglio Preiser CM, Krajewski S, Song K, Macdonald JS, Stemmermann G and Reed JC (1996a) Immunohistochemical analysis of Bcl-2 family proteins in adenocarciomas of the stomach. *Am J Pathol* **149**: 1449–1457

Krajewska M, Krajewski S, Epstein JI, Shabaik A, Sauvageot J, Song K, Kitada S and Reed JC (1996b) Immunohistochemical analysis of bcl-2, bax, bcl-X, and mcl-1 expression in prostate cancers. *Am J Pathol* **148**: 1567–1576

Krajewski S, Blomqvist C, Franssila K, Krajewska M, Wasenius VM, Niskanen E, Nordling S and Reed JC (1995) Reduced expression of proapoptotic gene Bax is associated with poor response rates to combination chemotherapy and shorter survival in women with metastatic breast adenocarcinoma. *Cancer Res* **55**: 4471–4478

Lu Q.1, Elia G, Lucas S and Thomas JA (1993) Bcl-2 proto-oncogene expression in Epstein–Barr-vrius-associated nasopharyngeal carcinoma. *Int J Cancer* **53**: 29–35

McDonnel TJ, Troncoso P, Brisbay SM, Logothetis C, Chung LWK, Hsieh JT, Tu SM and Campbell ML (1992) Expression of the proto-oncogene bcl-2 in the prostate and its association with emergence of androgene-independent prostate cancer. *Cancer Res* **52**: 6940–6944

Oltvai ZN, Milliman CL and Korsmeyer SJ (1993) Bcl-2 heterodimerizes in vivo with a conserved homolog, Bax, that accelerates programmed cell death. *Cell* **74**: 609–619

Paraf F, Gogusev J, Chretien Y and Droz D (1995) Expression of bcl-2 oncoprotein in renal cell tumours. *J Pathol* **177**: 247–252

 *© 2001 Cancer Research Campaign*

Pezzella F, Turley H, Kuzu I, Tungekar MF, Dunnil MS, Pierce CB, Harris A, Gatter KC and Mason DY (1993) bcl-2 protein in non-small-cell lung carcinoma. *N Engl J Med* **329**: 690–694

Re GG, Hazen-Martin DJ, El-Bahtimi R, Brownlee NA, Willingham MC and Garvin AJ (1999) Prognostic significance of Bcl-2 in Wilms' tumors and oncogenic potential of Bcl-$X_L$ in rare tumour cases. *Int J Cancer* **84**: 192–200

Reed JC (1994) Bcl-2 and the regulation of programmed cell death. *J Cell Biol* **124**: 1–6

Rieger L, Weller M, Bornemann A, Schabet M, Dichgans J and Meyermann R (1998) Bcl-2 family protein expression in human malignant glioma: a clinical-pathological correlative study. *J Neurol Sci* **155**: 68–75

Sinicrope FA, Hart J, Michelassi F and Lee JJ (1995) Prognostic value of Bcl-2 oncoprotein expression in stage II colon carcinoma. *Clin Cancer Res* **1**: 1103–1110

Strasser A, Harris AW, Bath ML and Cory C (1990) Novel primitive lymphoid tumours induced in transgenic mice by cooperation between myc and bcl-2. *Nature* **348**: 331–333

Tanaka K, Granata C, Want Y, O'Brian DS and Puri P (1999) Apoptosis and bcl-2 oncogene expression in Wilms' tumor. *Pediatr Surg Int* **15**: 243–247

Vaux DL (1993) Toward an understanding of the molecular mechanisms of physiological cell death. *Proc Natl Acad Sci USA* **90**: 786–789

Vaux DL, Cory S and Adams JM (1988) Bcl-2 gene promotes hematopoietic cell survival and cooperates with c-myc to immortalize pre-B cells. *Nature* **335**: 440–442

Xie X, Clausen OPF, De Angelis P and Boysen M (1998) Bax expression has prognostic significance that is enhanced when combined with AgNOR counts in glottic carcinomas. *Br J Cancer* **78**: 100–105

*© 2001 Cancer Research Campaign*
